# Unlocking the Potential of Arginine Deprivation Therapy: Recent Breakthroughs and Promising Future for Cancer Treatment

**DOI:** 10.3390/ijms241310668

**Published:** 2023-06-26

**Authors:** Yu-De Chu, Ming-Wei Lai, Chau-Ting Yeh

**Affiliations:** 1 Liver Research Center, Chang Gung Memorial Hospital, Linkou Branch, Taoyuan 333, Taiwan; yudechu@cgmh.org.tw; 2 Department of Pediatrics, Chang Gung Memorial Hospital, Linkou Branch and Chang Gung University College of Medicine, Taoyuan 333, Taiwan; 3 Molecular Medicine Research Center, College of Medicine, Chang Gung University, Taoyuan 333, Taiwan

**Keywords:** arginine deprivation cancer therapy, ADI-PEG 20, rhArg1-PEG, BCT-100, arginine auxotrophic cancer, circulating arginine, biomarker

## Abstract

Arginine is a semi-essential amino acid that supports protein synthesis to maintain cellular functions. Recent studies suggest that arginine also promotes wound healing, cell division, ammonia metabolism, immune system regulation, and hormone biosynthesis—all of which are critical for tumor growth. These discoveries, coupled with the understanding of cancer cell metabolic reprogramming, have led to renewed interest in arginine deprivation as a new anticancer therapy. Several arginine deprivation strategies have been developed and entered clinical trials. The main principle behind these therapies is that arginine auxotrophic tumors rely on external arginine sources for growth because they carry reduced key arginine-synthesizing enzymes such as argininosuccinate synthase 1 (ASS1) in the intracellular arginine cycle. To obtain anticancer effects, modified arginine-degrading enzymes, such as PEGylated recombinant human arginase 1 (rhArg1-PEG) and arginine deiminase (ADI-PEG 20), have been developed and shown to be safe and effective in clinical trials. They have been tried as a monotherapy or in combination with other existing therapies. This review discusses recent advances in arginine deprivation therapy, including the molecular basis of extracellular arginine degradation leading to tumor cell death, and how this approach could be a valuable addition to the current anticancer arsenal.

## 1. Introduction

Amino acids are vital components for protein synthesis in mammalian cells, particularly in rapidly dividing cells such as those in malignant tumors [1,2]. However, their functions extend beyond protein synthesis and encompass various physiological events, including the regulation of signaling transduction, gene expression, antioxidative responses, metabolic reprogramming, and immunity [3]. Notably, cancer cells often exhibit an auxotrophy to specific amino acids, which has been considered a hallmark in several cancer types [4,5]. This results in their dependency on the uptake of exogenous amino acids to meet the demands for rapid growth and metabolic processes. This phenotype has led to the development of a novel therapeutic concept of systemic amino acid deprivation, which aims to limit the supply of specific amino acids to cancer cells and consequently impair their growth and proliferation [6,7]. Therefore, amino acid deprivation therapy has emerged as a promising approach for the treatment of cancer.

Arginine is a versatile and essential amino acid that plays a crucial role in a wide range of biological functions. It is involved in various processes, including protein synthesis, cell proliferation, cell signaling, muscle contraction, immune defense, neurotransmission, vasodilation, growth factor regulation, and the synthesis of other amino acids [8,9]. Moreover, arginine plays a significant role in protein post-translational modification and immunomodulation [10,11]. Arginine can be obtained from both the diet and endogenous synthesis. It is predominantly synthesized through the de novo synthesis pathway known as the intestinal–renal axis. This pathway involves a collaborative effort between the small intestine and the kidney [12,13]. The epithelial cells of the small intestine break down dietary proteins, releasing citrulline into the bloodstream. Citrulline is then transported to the proximal tubule cells of the kidney, where it is converted to arginine through the urea cycle and sent back into circulation, serving as an extracellular arginine supply [14]. In addition to renal cells, many other cell types can also synthesize arginine. However, the de novo arginine synthesis pathway cannot fully meet metabolic demands under certain specific circumstances, such as those of infants in early development, children in rapid growing ages, and patients with severe inflammation, catabolic stress, and kidney or intestinal dysfunction [15,16]. Therefore, arginine is considered a semi-essential or conditionally essential amino acid.

**Figure 1:**
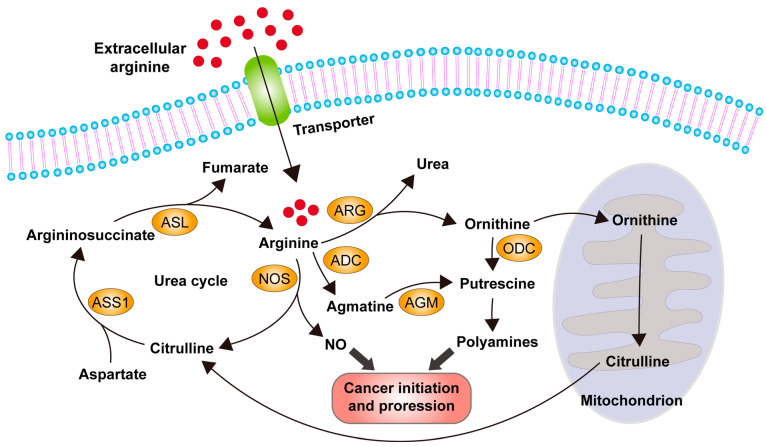
The diagram illustrates the metabolic pathways of arginine in human cells. Normally, free arginine can be obtained from extracellular sources through transporters to enter the cell, or it can be synthesized de novo from citrulline and aspartate in the urea cycle. Arginine can be further metabolized into nitric oxide (NO), urea, agmatine, and ornithine. Agmatine and ornithine serve as precursors for putrescine and downstream metabolites, polyamines. Ornithine can also be transported into the mitochondria for further processing to generate citrulline, which is then exported from the mitochondria and enters the urea cycle. Both NO and polyamines have been implicated in cancer initiation and progression and are considered key metabolites. Abbreviation: ASS1, argininosuccinate synthetase 1; ASL, argininosuccinate lyase; ADC, arginine decarboxylase; AGM, agmatinase; ODC, ornithine decarboxylase; ARG, arginase; NOS, nitric oxide synthase.

Arginine is involved in various metabolic pathways, including the production of nitric oxide (NO), urea, agmatine, and ornithine. Agmatine and ornithine can serve as precursors of putrescine, which is the key precursor of downstream metabolites polyamines. Both NO and polyamines are considered to be crucial metabolites involved in cancer initiation, invasion, metastasis, and angiogenesis through various signaling pathways. In healthy individuals, arginine can be obtained through the urea cycle, where citrulline is converted into arginine. Tissue cells outside the kidney can also synthesize arginine through the urea cycle. Intracellular arginine can be directly acquired from circulation, where it is imported into the cell through an arginine transporter, or it can be obtained by de novo synthesis from citrulline and aspartate in the urea cycle. Cytoplasmic ornithine can be transported into the mitochondrion for further processing to generate citrulline, which is then exported from the mitochondrion and enters the urea cycle for arginine biosynthesis (Figure 1) [13,17,18].

The key enzymes involved in arginine biosynthesis through the urea cycle are arginosuccinate synthase 1 (ASS1) and arginosuccinate lyase (ASL) [19]. However, in many cancer cells, the expression levels of ASS1 are significantly downregulated, leading to an arginine auxotrophic phenotype and a high dependency on extracellular arginine supply. Therefore, the deprivation of extracellular arginine supply has emerged as a potential therapeutic strategy to treat cancers with arginine auxotrophy [8]. Several strategies have been designed to achieve arginine deprivation for anticancer therapy and have entered clinical trials, demonstrating sustained therapeutic efficacy in cancer patients [20,21,22].

## 2. Anticancer Treatments Using Arginine Deprivation Therapy

As described in the previous section, arginine is involved in the biosynthesis of nitric oxide, agmatine, and polyamines, which activate signaling pathways regulating cell growth, invasion, and metastasis in cancer cells [13,17,18]. Given its high demand in tumor growth and progression, arginine represents an attractive target for anticancer therapy.

**Figure 2:**
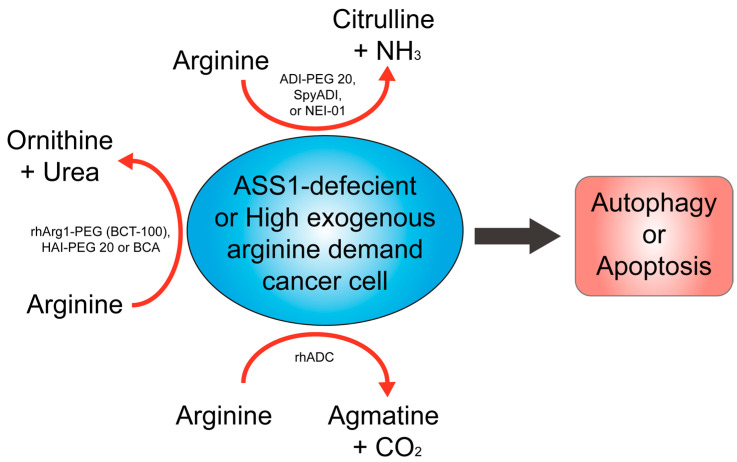
Concept of using arginine-depleting enzymes in arginine deprivation for cancer treatment. Cancer cells that lack the ASS1 enzyme and require external sources of arginine are susceptible to this therapy. Three categories of arginine-depleting enzymes, including arginine deiminase related (ADI-PEG 20, SpyADI, NEI-01), arginase related (rhArg1-PEG, HAI-PEG 20 or BCA), and arginine decarboxylase related (rhADC), can be used to block the external supply of arginine, leading to cancer cell death through autophagy or apoptosis. ADI, arginine deiminase; SpyADI, *Streptococcus* pyogenes ADI; Arg, arginase; HAI-PEG 20, human arginase 1 mutant-PEG 20; BCA, Bacillus Caldovelox arginase; ASS1, argininosuccinate synthetase 1.

Arginine deprivation therapy can selectively target cancer cells with arginine auxotrophy, where the cancer cells, but not the healthy cells, exhibit decreased expression of the ASS1 enzyme. ASS1 is a rate-limiting enzyme in the urea cycle that synthesizes arginine from citrulline [20,21,22]. As a result, these cancer cells depend on extracellular arginine supply to sustain their growth and proliferation, and arginine deprivation kills the cells. This approach can be achieved using various agents, including arginine-degrading enzymes, such as arginine deiminase (ADI), arginase, and arginine decarboxylase. These enzymes convert arginine to citrulline, ornithine, or agmatine, respectively, thereby depriving cancer cells of their essential amino acid (see following sections and Figure 2).

Some types of cancer, such as the ones listed in Table 1, exhibit downregulation of the ASS1 enzyme, making them suitable for the use of arginine deprivation therapy. Researchers have determined the expression levels of ASS1 in these cancer types using various methods, including cDNA dot blotting, immunohistochemical staining, Western blotting, real-time quantitative PCR, and/or cDNA microarray (Table 1). This evidence suggests that the use of arginine deprivation therapy to selectively deprive cancer cells of arginine could be a favorable strategy for these cancer types.

## 3. Enzyme-Mediated Arginine Deprivation Agents for Cancer Therapy: Pre-Clinical and Clinical Evidence

Arginine deprivation therapy aims to reduce the extracellular supply of arginine in tumors with an arginine auxotrophic phenotype. Various agents can achieve this goal (as shown in Figure 2). However, the in vivo application of arginine-depleting enzymes faces challenges, such as the short circulating half-life in plasma and high immunogenicity [38]. To overcome these problems, one strategy is to covalently link a well-known chemical modifier, polyethylene glycol (PEG), to the amino group of the proteins, providing advantages, such as low antigenicity, low toxicity, and an extended circulating half-life [39]. Another strategy is to genetically fuse therapeutic proteins with an antibody Fc domain or serum albumin, which prolongs their circulation time and prevents lysosomal degradation by binding to Fc receptors (FcRn) in cells [40].

Currently, researchers have developed and isolated four independent arginine catabolic enzymes, including microbial-derived ADI, recombinant human arginase 1 (rhArg1), recombinant human arginine decarboxylase (rhADC), and Bacillus Caldovelox arginase (BCA). The current status of each of these agents is described and discussed in the following sections.

### 3.1. ADI

#### 3.1.1. Preclinical Evidence Supporting ADI as a Potential Strategy for Cancer Therapy

ADI (E.C.3.5.3.6) is an enzyme found in prokaryotes that irreversibly converts arginine to citrulline and ammonium ion, thereby limiting the availability of exogenous arginine to cancer cells [41]. Originally purified, cloned, and sequenced from *Mycoplasma* [42,43,44], researchers recognized the potential of arginine deprivation therapy for anticancer treatment in the late 1990s and early 2000s, leading to the development of ADI (see reference [8] for a review). To reduce the immunogenicity of bacterial-originated ADI in humans, ADI-PEG 20, a PEGylated form of ADI with increased half-life and stability, was developed [45,46]. Preclinical studies and clinical trials have shown that ADI-PEG 20, in combination with other therapies, has encouraging results for the treatment of various cancers [8]. In preclinical studies, ADI-PEG 20 has demonstrated efficacy against various arginine auxotrophic cancers (see below for details).

Research has indicated that in the treatment of glioma, sensitivity to arginine deprivation can be predicted by the methylation of neoplasia-specific CpG islands in the ASS1 and ASL genes, using ex vivo cultures and cell lines [47,48]. Combining ADI-PEG 20 with radiotherapy was found to result in complete radiological and pathological response, as well as extended disease-free survival, with no significant toxicity observed in a glioma mouse model in vivo [47,48]. Additionally, ADI-PEG 20 was shown to reduce intracranial growth of ASS1-negative glioma, extend mouse survival, and enhance the effects of temozolomide in both ASS1-negative and -positive backgrounds, suggesting its potential as a biomarker for glioma treatment [49,50].

ADI-PEG 20 has shown efficacy in liver cancer, particularly in hepatocellular carcinoma (HCC), due to the absence of ASS1 in cell lines [51]. In vitro and in vivo studies showed that ADI-PEG 20 treatment restrained the growth of arginine-dependent mouse and human HCC cell lines. Additionally, ADI-PEG 20 has shown effectiveness in treating cisplatin-resistant HCC cell lines, and a combination treatment with ADI-PEG 20 and 5-Fluorouracil was effective in reducing cell viability and increasing apoptosis in HCC cell lines with low levels of ASS1 [52,53]. Moreover, a study indicated that the expression levels of WW-domain-containing oxidoreductase (WWOX) and hypoxia-inducible factor-1 (HIF-1) may impact the susceptibility of HCC cells to ADI-PEG 20, implying the potential use of these proteins as biomarkers for ADI-PEG 20 in liver cancer treatment [28].

ADI-PEG 20 has shown promise in pancreatic cancer treatment, particularly by inhibiting the growth of xenografts lacking ASS1 expression and inducing cell apoptosis in pancreatic cancer cell lines [54]. When combined with gemcitabine, ADI-PEG 20 significantly increased tumor response and inhibited gemcitabine-induced overexpression of ribonucleotide reductase regulatory subunit M2 (RRM2) levels, a key regulator of gemcitabine efficacy associated with resistance in both cell lines and xenograft mouse models [55]. ASS1-low tumors in pancreatic cancer patients have been associated with a poor prognosis, suggesting that arginine deprivation therapy might play a role in treating pancreatic cancer [29]. The combination of ADI-PEG 20, gemcitabine, and docetaxel was shown to be effective in inhibiting tumor growth and rendering cells susceptible to gemcitabine in pancreatic cancer cell lines [56]. In addition, ADI-PEG 20 was found to potentiate the radio-sensitivity of ASS1-deficient pancreatic cancer cells, leading to apoptosis [57]. Furthermore, pano-binostat, a non-selective histone deacetylase inhibitor, and ADI-PEG 20 were effective in suppressing ASS1-low pancreatic cancer growth in mouse xenograft models [58]. A novel recombinant miR-1291 agent also demonstrated effectiveness in sensitizing pancreatic cancer cells to ADI-PEG 20 and improved cisplatin efficacy in inhibiting pancreatic cancer cell viability [59].

ADI-PEG 20 shows potential as a therapeutic drug for prostate cancer, especially in cell lines with reduced ASS1 expression. Autophagy is induced by ADI-PEG 20, but the inhibition of autophagy accelerates ADI-PEG 20-induced cell death in vitro [60]. Combining ADI-PEG 20 with docetaxel inhibits tumor growth in vivo, suggesting that targeting multiple cell death pathways may provide additional avenues for cancer treatment [61]. Prolonged arginine depletion leads to “chromatophagy”, which is characterized by giant autophagosomes, nucleus membrane rupture, and histone-associated DNA leakage due to mitochondrial damage and increased ROS production in prostate cancer cell lines [62]. Drug resistance development poses a challenge in arginine-deprivation therapy. Recent research has uncovered a link between aberrant activation of the immunoglobulin superfamily transmembrane protein, triggering the receptor expressed on myeloid cells 1 (TREM1), and the chemokine (C-C motif) ligand 2 (CCL2) axis. Interestingly, this activation was found to coincide with the activation of the CCL2 pathway, leading to the activation of extracellular signal-regulated kinase 1/2 (ERK1/2), AKT serine/threonine kinase (AKT), and the mechanistic target of rapamycin kinase (mTOR), which are crucial for cell proliferation, and the signal transducer and activator of transcription 3 (STAT3), which contributes to a positive feedback loop of CCL2 expression, ultimately promoting cell survival and drug resistance. Knockdown of TREM1 or CCL2 sensitizes ADI-PEG 20-resistant cells to treatment in vitro, providing novel strategies to overcome resistance [63].

Breast cancer cells lacking ASS1 undergo autophagy-dependent death when subjected to arginine starvation. ADI-PEG 20 treatment leads to prolonged arginine starvation and results in mitochondrial oxidative stress, leading to cytotoxic autophagy in breast cancer cell lines. ADI-PEG 20-based arginine starvation therapy has the potential to become a treatment option for breast cancer patients with low or absent levels of ASS1 [64]. Furthermore, researchers developed a pH-sensitive cell-penetrating peptide-based delivery system to transport ADI-PEG 20 into the cells, which shows better selectivity towards tumor cells. This approach may help to overcome ADI-PEG 20 resistance caused by hypoxia in breast cancer cells [65].

Treating sarcoma is challenging, as standard chemotherapy often shows a limited response, and there are no common gene mutations or alterations. However, studies have identified metabolic vulnerabilities, including the downregulation of ASS1 in patients with myxofibrosarcoma, which correlates with increased tumor grade and stage [34,66]. The arginine-depriving agent ADI-PEG 20 was investigated as a treatment for sarcoma [34,56,66]. Although ADI-PEG 20 alone did not induce cell death, the addition of chloroquine resulted in sarcoma cell death in vitro [34]. Therefore, targeting metabolic vulnerabilities could offer alternative treatment strategies for sarcoma, highlighting the importance of considering metabolic defects in cancer treatment [67]. Positron emission tomography (PET) tracers, such as ^18^F-labeled histidine analogue, (*S*)-2-amino-3-[1-(2-[^18^F]fluoroethyl)-1*H*-[1,2,3]triazol-4-yl]propanoic acid ([^18^F]AFETP), were used to monitor the response to arginine starvation induced by ADI-PEG 20 [68], while a biosensor was developed to monitor arginine-dependent protein translation in ASS1-deficient sarcoma cancer cells in vitro [69]. These findings suggest that targeting metabolic vulnerabilities, such as ASS1 downregulation, could provide alternative treatment strategies for sarcoma. Furthermore, using PET tracers and biosensors could monitor the response to metabolically targeted therapies.

Standard chemotherapy for epithelial ovarian cancer does not take into consideration the varying responses among different histologic subtypes. However, arginine depletion therapy with ADI-PEG 20 has shown potential for various ovarian cancer subtypes, including some non-serous ovarian cancer subtypes, due to significantly reduced ASS1 expression [70]. Another study identified potential therapeutic targets for rare ovarian cancer subtypes, such as small cell carcinoma of the ovary hypercalcemic type (SCCOHT), which are resistant to standard chemotherapy [35]. In vitro and in vivo studies demonstrated the effectiveness of ADI-PEG 20 in inhibiting tumor growth in SCCOHT cell line-based mouse xenograft models and patient-derived mouse xenograft models [35]. Thus, clinical trials with ADI-PEG 20 in non-serous ovarian carcinoma subtypes and SCCOHT are warranted to investigate its efficacy further.

ADI-PEG 20 has the potential to inhibit human melanoma growth in vitro by degrading extracellular arginine. However, its short half-life in vivo limits its efficacy, which has been addressed by developing a longer half-life formulation [51]. ADI-PEG 20 can induce transcriptional activation of the rate-limiting enzyme ASS1 in some melanoma cell lines and combining it with TRAIL enhances apoptosis and accelerates cell death in vitro [71,72,73,74]. Drug resistance remains a challenge, and alternative PET tracers targeting apoptotic pathway activation, cell death and plasma nitric oxide levels may be useful in evaluating clinical responses in melanoma [75,76,77]. Combining ADI-PEG 20 with cisplatin improves therapeutic efficacy in melanoma cell lines through increased DNA damage [78]. Further investigation is necessary to optimize ADI-PEG 20 efficacy and overcome drug resistance. Monitoring the tumor condition with alternative PET tracers and quantifying tumor vascularization may be useful. Combining ADI-PEG 20 with other drugs may enhance therapeutic efficacy.

Numerous studies have explored treatment options for malignant pleural mesothelioma (MPM). One study proposed a unique approach using targeted molecular therapeutics based on selected tumor markers [79]. The study demonstrates that MPM cells lack the ability to synthesize arginine and can be targeted with ADI-PEG 20, with increased caspase-3 activation and cell death in vitro. TRAIL can further amplify the apoptotic signal by cleaving pro-apoptotic protein BH3-interacting-domain death agonist (BID). ADI-PEG 20 induces selective lethality in ASS1-negative MPM cells. However, ADI-PEG 20 resistance can occur through ASS1 re-expression via demethylation of the promoter. The study using in vitro assays identified a synthetic lethal dependence between ASS1 deficiency and polyamine metabolism, which could be exploited for treating ASS1-negative cancers [80]. Another study investigated the role of nuclear deubiquitylase BRCA1-associated protein 1 (BAP1) in MPM cell physiology and drug sensitivity [81]. BAP1 is frequently inactivated in MPM, and germline BAP1 mutation predisposes to some cancers, including MPM. The study reveals metabolic adaptation and an inter-relationship between BAP1 and arginine metabolism. BAP1-negative/ASS1-expressing MPM cell lines are more sensitive to ASS1 inhibition, which implies that epithelioid MPM patients will likely benefit from ADI-PEG 20 treatment [81]. These studies provide a foundation for designing clinical trials for MPM.

ADI-PEG 20 has also shown promise as a therapeutic approach in other cancers. In small cell lung cancer (SCLC), ADI-PEG 20 induced cell death in ASS1-deficient cells and inhibited tumor growth in ASS1-negative xenografts [45,82]. Similarly, in head and neck cancer, ADI-PEG 20 inhibited cancer cell growth in vitro, and high tumor ASS1 status predicted poor disease-free survival in oral squamous carcinoma patients [27]. In renal cell carcinoma, ADI-PEG 20 effectively treated cells with low levels of intracellular ASS1, causing significant growth retardation and prolonged survival in tumor-bearing mice [30]. In hereditary leiomyomatosis and renal cell cancer, ADI-PEG 20 depleted arginine and decreased cell survival and proliferation in fumarate dehydratase (FH)-deficient cells [83]. In bladder cancer, ADI-PEG 20 was synthetically lethal in ASS1-methylated bladder cells in vitro, and a decrease in intracellular thymidine levels was observed [32]. Furthermore, ADI-PEG 20 selectively arrested tumor growth in mice with ASS1-deficient bladder cancer cells [31]. In lymphoma and leukemia, preclinical studies have shown that ADI-PEG 20 has the potential to serve as a treatment option [36,37,84]. The studies suggest that ADI-PEG 20 can induce caspase activation in sensitive acute myeloid leukemia (AML) cell lines, resulting in responses in all AML cell lines tested in vivo, when combined with cytarabine chemotherapy [37]. Additionally, ADI-PEG 20 efficiently increased the anticancer effect of dexamethasone on human leukemic CEM cells through G1-cell-cycle arrest in vitro [84]. These studies suggest that ADI-PEG 20 has the potential to serve as a treatment option for lymphoma and leukemia, and further studies are warranted to explore its therapeutic applications.

In summary, ADI-PEG 20 shows great promise as a therapeutic approach for many of the cancer types mentioned above. Its efficacy can be attributed to its ability to induce cell death in cancer cells that lack the essential enzyme ASS1 for arginine synthesis. Furthermore, ADI-PEG 20 has been demonstrated to augment the efficacy of other chemotherapy agents and radiotherapy, offering a potential strategy for combination therapy. Although further clinical trials are required to confirm these findings, ADI-PEG 20 has the potential to serve as a therapeutic agent for various cancer types.

SpyADI, a different origin of ADI isolated from *Streptococcus* pyogenes, has shown potential in treating glioma as a monotherapy, with additive or synergistic effects when combined with other drugs. However, its immunogenicity and stability in vivo are to be addressed by PEGylation. Combining SpyADI with CDK inhibitors resulted in synergistic antitumoral effects, inducing autophagy, mitochondrial and endoplasmic reticulum damage, and suppression of the DNA damage repair system via non-homologous end-joining and homologous repair. Combining SpyADI with Mithramycin A was found to be superior to sequential application, resulting in the enhanced impairment of colony formation, induction of senescence, and an increase iin mitochondrial membrane polarization and autophagy in glioma cells in vitro. Despite its promise for glioma treatment, more research is needed to evaluate its efficacy, safety, and dosing on other cancer types [85,86,87,88,89].

NEI-01 is another novel ADI-based drug that selectively depletes arginine in tumor cells to induce metabolic stress and cell death. NEI-01 has greater specificity for cancer cells and a longer half-life due to its albumin-binding domain [90]. In preclinical studies, NEI-01 showed potent anticancer activity against different types of cancer, including pancreatic cancer and leukemia [90,91]. NEI-01 treatment significantly reduced tumor volume and weight in a human pancreatic mouse xenograft model and demonstrated potent anti-leukemia activities in three different types of mouse models [90,91]. Currently, a phase I clinical trial (NCT05226468) of NEI-01 in patients with advanced solid tumors and lymphoma is ongoing, which may represent a potential new approach to arginine deprivation therapy for cancer treatment.

#### 3.1.2. Overview of Clinical Trials Using ADI-PEG 20 for Anticancer Therapy and Their Outcomes

As of now, there are 29 clinical trials that have been registered and are publicly searchable in the National Clinical Trial database, either ongoing or terminated (refer to Table 2). These trials are based on the encouraging results of pre-clinical studies, which included various cancer types, such as melanoma, HCC, SCLC, non-small cell lung cancer (NSCLC), MPM, prostate cancer, ovarian cancer, breast cancer, pancreatic cancer, AML, NHL, glioblastoma multiforme (GBM), and sarcoma. The results of these trials have been published and are described and discussed below.

A phase I clinical trial was conducted to assess the safety and preliminary efficacy of ADI-PEG 20 in combination with pemetrexed and cisplatin for heavily pretreated patients with recurrent high-grade gliomas (HGG) harboring epimutations of ASS1 and/or ASL [78]. Ten patients received ADI-PEG 20 plus pemetrexed and cisplatin (ADIPemCis) therapy, and the outcomes showed that it was well-tolerated with no severe adverse events. Stable disease was achieved in 80% of patients, and plasma arginine levels were significantly suppressed while citrulline levels increased during the sampling period. The median progression-free survival was 5.2 months, and overall survival was 6.3 months. The study concluded that ADIPemCis was well-tolerated and demonstrated favorable results compared to historical controls. Further trials of ADI-PEG 20 in HGG are planned [92].

ADI-PEG 20 has undergone several clinical trials for advanced solid malignancies. In a phase I study with docetaxel, effective clinical activity was observed with only one case of dose-limiting toxicity reported at the lowest dose level [93]. Another phase I study with cisplatin and pemetrexed found the treatment to be well-tolerated with no dose-limiting toxicities reported and clinical activity observed in poor-prognosis tumors [94]. In a phase II study of patients with ASS1-deficient SCLC, stable disease was observed in 18.2% of patients, and the treatment was well-tolerated with no unexpected adverse events or discontinuations [95]. Lastly, a phase I dose-expansion trial in NSCLC patients with ASS1 deficiency found that ADI-PEG 20 was well-tolerated, and the treatment resulted in a disease control rate of 85.7% and a partial response rate of 47.6% [96].

ADI-PEG 20 has undergone several clinical trials for the treatment of HCC. One trial treated a patient with unresectable HCC with escalating doses of ADI-PEG 20, resulting in a reduction in plasma arginine levels, tumor size, and serum alpha-fetoprotein levels, with no treatment-related toxicities or side effects [97]. Another study, a phase I/II cohort dose-escalation trial, found that ADI-PEG 20 therapy was effective and well-tolerated in some HCC patients, with two complete responses, seven partial responses, and seven cases of stable disease reported out of 19 patients [98]. In HCC patients with HCV, ADI-PEG 20 selectively inhibited HCV replication in vitro and reduced HCV titers by up to 99% in 50% of HCV-serotype 1b patients, leading to significant improvements in liver function [99]. ADI-PEG 20 was also found to be safe and effective in stabilizing disease progression in heavily pretreated advanced HCC patients, although there were no objective responders [100,101]. The combination of ADI-PEG 20 and modified FOLFOX6 was safe and effective in treating advanced HCC, with an objective response rate of 21% and a median progression-free survival of 7.3 months and overall survival of 14.5 months [102]. However, a phase III study did not show a significant difference in overall survival or progression-free survival between ADI-PEG 20 and placebo [103]. Another phase II study found ADI-PEG 20 to be safe and effective in stabilizing the progression of heavily pretreated advanced HCC in an Asian population, while a study evaluating the combination of ADI-PEG 20 and modified 5-fluorouracil, leucovorin, and oxaliplatin (mFOLFOX6) in patients with advanced HCC who had progressed on at least two prior lines of treatment exhibited limited antitumor activity [104].

ADI-PEG 20 has been studied in several clinical trials for the treatment of melanoma. A phase I/II study enrolled 15 patients in the US and 24 patients in Italy and used ADI-PEG 20 to lower plasma arginine levels in individuals with metastatic melanoma [105]. In the Italian cohort, six patients responded to the treatment, resulting in a 25% response rate and prolonged survival. Another study explored the relationship between ASS1 expression in melanoma tumors and response to arginine-depleting therapy with ADI-PEG 20. The results showed that ADI-PEG 20 is safe and effective in melanoma patients with negative ASS1 expression, while ASS1 positivity is associated with drug resistance and tumor progression [106]. In a phase I/II study, ADI-PEG 20 was given to 31 previously treated patients with advanced melanoma. Although no objective responses were observed, nine patients achieved stable disease, and pharmacodynamic analysis revealed complete plasma arginine depletion in 30 out of 31 patients [107]. In a phase 1 trial, the safety and efficacy of the combination of ADI-PEG 20 and cisplatin were evaluated in patients with metastatic ASS1-deficient solid tumors. The combination had an acceptable safety profile and showed antitumor activity, with 5% of patients experiencing partial responses and 41% having stable disease [108]. A pilot study investigated the safety and tolerability of a triple combination therapy consisting of ADI-PEG 20 and immunotherapy in patients with metastatic uveal melanoma, which was safe and tolerable but did not result in any objective responses [109]. Finally, a phase I dose-expansion study assessed the safety and preliminary activity of ADI-PEG 20 combined with pemetrexed and cisplatin chemotherapy in patients with metastatic uveal melanoma and ASS1 deficiency. Although seven patients had stable disease with a median progression-free survival of 3.0 months and a median overall survival of 11.5 months, tumor re-biopsies at progression revealed ASS1 re-expression as an escape mechanism, indicating the need for further investigation of arginine deprivation in uveal melanoma [110].

Several clinical studies have investigated the effectiveness of ADI-PEG 20 for treating MPM. In one phase I trial, nine chemotherapy-naïve patients with MPM or non-small cell lung cancer received varying doses of ADI-PEG 20 along with pemetrexed and cisplatin. Seven of the nine patients showed partial responses, and no dose-limiting toxicities were reported. The study recommended a dose of weekly ADI-PEG 20 36 mg/m^2^ plus three-weekly cisplatin 75 mg/m^2^ and pemetrexed 500 mg/m^2^ for future studies [94]. The ADAM study, a multicenter phase II randomized clinical trial, demonstrated that arginine deprivation with ADI-PEG 20 improved progression-free survival in patients with ASS1-deficient mesothelioma without significant adverse events. The study indicated that targeting arginine is safe and warrants further clinical investigation in arginine-dependent cancers [111]. Additionally, a phase 1 dose-expansion study of ADI-PEG 20 in combination with pemetrexed and cisplatin was conducted with 32 chemotherapy-naïve patients with ASS1-deficient malignant pleural mesothelioma. The treatment was well-tolerated, and the disease control rate was 93.5%, with a partial response rate of 35.5%. The median progression-free survival and overall survival were 5.6 and 10.1 months, respectively. Biopsies revealed that resistance to ADI-PEG 20 correlated with macrophage recruitment and tumor immune microenvironment. These results support the ongoing phase III study of ADIPemCis for the treatment of mesothelioma [112]. It is worth noting that the final report of a national multicenter phase II/III clinical trial recruiting patients with MPM (NCT02709512) has met its endpoints with significantly increased overall survival and progression-free survival. Although detailed results are pending, the company that produces ADI-PEG 20 is now contacting the Food and Drug Administration (FDA) for fast-track designation.

At present, only two clinical trials recruiting patients with lymphoma or leukemia have investigated the efficacy of ADI-PEG 20 treatment, with summarized reports available. In a phase II study evaluating ADI-PEG 20 in relapsed/refractory/poor-risk AML patients, the disease control rate was 42.9%, with complete responses observed in 2 (9.5%) patients and stable disease in 7 (33.3%) of the 21 evaluable patients. The duration of response for the two patients with complete responses was 7.5 and 8.8 months. The study found that ASS1 deficiency was necessary but insufficient for response to ADI-PEG 20 monotherapy in AML, and further biomarkers and mechanistic explorations are critical for identifying appropriate patients for future trials [113]. In another phase I trial investigating the safety and efficacy of combining ADI-PEG 20 and low-dose cytarabine in patients with AML, the combination therapy was well-tolerated and resulted in an overall response rate of 44.4%, with a median overall survival of eight months. The overall response rate was higher in treatment-naïve patients (71.4%), suggesting that further combination studies of ADI-PEG 20 and low-dose cytarabine are warranted [114]. These trials demonstrate the potential of ADI-PEG 20 in treating lymphoma and leukemia, but more research is needed to identify predictive biomarkers and understand the underlying mechanisms of action.

ADI-PEG 20 has also been investigated in patients with metastatic pancreatic cancer and breast cancer. A phase 1/1B study was conducted to determine the maximum tolerated dose and recommended a phase II dose of ADI-PEG 20 in combination with nab-paclitaxel and gemcitabine. The study involved two cohorts of patients, and no dose-limiting toxicities were observed in cohort 1. Cohort 2 was expanded to six patients after one occurrence of dose-limiting toxicity. The recommended phase 2 dose of ADI-PEG 20 was 36 mg/m^2^ weekly when combined with standard-dose gemcitabine and nab-paclitaxel. The study demonstrated that the overall response rate in patients treated with this dose was 45.5% in the first-line setting. The treatment was found to be active in previously treated and untreated patients with advanced pancreatic cancer, including those with ASS1-deficient and -proficient tumors [115]. In another phase I study, the safety and tolerability of combining ADI-PEG 20 with liposomal doxorubicin in patients with metastatic solid tumors. Of 15 patients enrolled, 9 had metastatic HER2-negative breast carcinoma. The trial followed a 3 + 3 design. No dose-limiting toxicities or treatment-related deaths were observed. Of the 15 patients, stable disease was achieved in 9 patients, and the overall median progression-free survival time was 3.95 months. The study concluded that the combination of ADI-PEG 20 and liposomal doxorubicin had an acceptable safety profile. Further evaluation of this combination is being discussed [116].

**Table 2:** Review of clinical trials assessing the therapeutic effectiveness of ADI-PEG 20 in cancer treatment.

Title	Status	Conditions	Interventions	Phases	Start Date	Enrollment	Outcome	NCT	Ref
ADI-PEG in Patients with Metastatic Melanoma	Completed	Melanoma	ADI-PEG 20	Phase I	2001/9/1	15	NA	NCT00029900	
Testing of ADI-PEG in Hepatocellular Carcinoma	Completed	HCC	ADI-PEG 20	Phase II	2002/9/1	34	NA	NCT00056992	
Pegylated Arginine Deiminase in Treating Patients with Metastatic Melanoma That Cannot Be Removed by Surgery	Completed	Melanoma	ADI-PEG 20	Phase II	2004/6/1	38	38 subjects were enrolled, 10 were ASS1 positive, and 17 were ASS1 negative. Median OS was 9.3 vs. 14.6 months for ASS1 positive vs. ASS1 negative, respectively. Median TTP was 1.8 vs. 3.6 months for ASS1 positive vs. ASS1 negative, respectively. 11 subjects who declined pre-treatment biopsies were not assessed.	NCT00450372	[106]
Study of ADI-PEG 20 in Patients with Advanced Melanoma	Completed	Melanoma	ADI PEG 20	Phase I/II	2007/7/1	31	No objective responses were seen, but 9 patients had SD. Pharmacodynamic analysis showed complete plasma arginine depletion in 30 of 31 patients.	NCT00520299	[107]
Study of ADI-PEG 20 in Patients with Relapsed Sensitive or Refractory Small Cell Lung Cancer	Terminated	SCLC	ADI-PEG 20	Phase II	2011/1/1	22	20 evaluable patients were included in two cohorts: 8 with “sensitive” disease and 12 with “refractory” disease. 2 out of 8 had SD in the “sensitive” cohort and 2 out of 12 had SD in the “refractory” cohort. Pharmacodynamic analysis showed complete plasma arginine depletion in 21 out of 22 patients.	NCT01266018	
A Clinical Trial of ADI-PEG 20TM in Patients with Malignant Pleural Mesothelioma	Unknown	MPM	ADI-PEG 20	Phase II	2011/1/1	70	68 patients were evaluated, with a median PFS of 3.2 vs. 2.0 months (*p* = 0.03) in the ADI-PEG 20 group vs. best supportive care group. There were no significant differences between groups in response rate, OS, and adverse event rate. The best response observed was SD.	NCT01279967	[111]
Ph 3 ADI-PEG 20 Versus Placebo in Subjects with Advanced Hepatocellular Carcinoma Who Have Failed Prior Systemic Therapy	Completed	HCC	ADI-PEG 20	Phase III	2011/7/1	636	635 patients were evaluated, with 70% progressing on sorafenib. Median OS was 7.8 months for ADI-PEG 20 and 7.4 months for placebo (*p* = 0.88, HR = 1.02), while median PFS was 2.6 months for both (*p* = 0.07, HR = 1.17). The death rate within 30 days of the end of treatment was 15.2% on ADI-PEG 20 and 10.4% on placebo, with none related to therapy. A trend towards improved OS was observed in patients with more prolonged arginine depletion.	NCT01287585	[103]
Ph 1 Trial of ADI-PEG 20 Plus Docetaxel in Solid Tumors with Emphasis on Prostate Cancer and Non-Small Cell Lung Cancer	Completed	Solid Tumors and Prostate Cancer	ADI-PEG 20	Phase I	2011/9/6	43	NA	NCT01497925	
Ph 1 Trial of ADI-PEG 20 Plus Cisplatin in Patients with Metastatic Melanoma	Completed	Melanoma and OC	ADI-PEG 20	Phase I	2012/9/1	8	NA	NCT01665183	
PH 2 ADI-PEG 20 Study in Non-Hodgkin’s Lymphoma Subjects Who Have Failed Prior Systemic Therapy	Completed	NHL	ADI-PEG 20	Phase II	2013/12/6	18	NA	NCT01910025	
Ph 1 ADI-PEG 20 Plus Doxorubicin; Patients with HER2 Negative Metastatic Breast Cancer	Completed	HER2(-ve) Breast Cancer	ADI-PEG 20	Phase I	2014/4/1	15	NA	NCT01948843	
Ph 1 Study in Subjects with Tumors Requiring Arginine to Assess ADI-PEG 20 With Pemetrexed and Cisplatin	Terminated	MPM, NSCLC, Melanoma, HCC, Glioma, and Sarcoma	ADI-PEG 20	Phase I	2014/4/23	85	Results were reported in 3 studies. In the first study, 7 patients had SD with a median PFS of 3.0 months and a median OS of 11.5 months. In the second study, the DCR was 85.7% with a PR rate of 47.6%. Median PFS and OS were 4.2 and 7.2 months, respectively. In the third study, treatment was well tolerated with a best overall response of SD in 8 patients. Median PFS was 5.2 months and OS was 6.3 months.	NCT02029690	[92,96,110]
Ph 2 Trial of ADI PEG 20 Plus Concurrent Transarterial Chemoembolization (TACE) vs. TACE Alone in Patients with Unresectable Hepatocellular Carcinoma	Completed	Unresectable HCC	ADI-PEG 20 + TACE	Phase II	2014/10/15	30	NA	NCT02006030	
Ph 1-2 Study ADI-PEG 20 Plus FOLFOX in Subjects with Advanced GI Malignancies Focusing on Hepatocellular Carcinoma	Terminated	Advanced GI cancer	ADI-PEG 20 + mFOLFOX6	Phase I/II	2014/11/1	140	In the phase 1 trial, 27 patients were enrolled (23 with advanced HCC and 4 with other GI tumors). No dose-limiting toxicities were observed in cohort 1 or 2, and the recommended phase 2 dose for ADI-PEG 20 was 36 mg/m^2^ weekly with mFOLFOX6. The ORR was 21% (95% CI 7.5–43.7), with a median PFS and OS of 7.3 and 14.5 months, respectively. Arginine levels were depleted despite low levels of anti-ADI-PEG 20 antibodies, but there was no correlation between arginine depletion at 4 and 8 weeks and archival tumoral ASS1 levels with response	NCT02102022	[102]
Ph 1 Trial of ADI PEG 20 Plus Sorafenib to Treat Patients with Liver Cancer	Completed	HCC	ADI-PEG 20	Phase I	2014/11/1	8	NA	NCT02101593	
Ph 1B Trial With ADI-PEG 20 Plus Nab-Paclitaxel and Gemcitabine in Subjects with Pancreatic Cancer	Completed	Pancreatic Cancer	ADI-PEG 20	Phase I	2014/11/17	21	NA	NCT02101580	
PH 2 ADI-PEG 20 Acute Myeloid Leukemia	Completed	AML	ADI-PEG 20	Phase II	2015/1/6	43	Among 21 evaluated patients, 2 (9.5%) achieved CR and 7 (33.3%) had stable disease, resulting in a DCR of 42.9%. The duration of response for the two CR patients was 7.5 and 8.8 months. DCR was associated with arginine depletion to ≤10 μM for a median of 8 weeks.	NCT01910012	[113]
Ph 1 Study of ADI-PEG 20 Plus Low Dose Cytarabine in Older Patients With AML	Terminated	AML	ADI-PEG 20 + Cytarabine	Phase I	2017/1/20	23	23 patients were given low-dose cytarabine subcutaneously twice daily for 10 days every 28 days and ADI-PEG20 at 18 or 36 mg/m^2^ (dose levels 1 and 2) intramuscularly weekly. 18 evaluable patients had a 44.4% ORR and median OS of 8.0 (4.5-not reached) months. In 7 treatment-naïve patients, the ORR was 71.4% with 57.1% complete remission rate. No dose-limiting toxicities were reported, and the combination was well-tolerated.	NCT02875093	[114]
Study ADI-PEG 20 Plus Pembrolizumab in Advanced Solid Cancers	Terminated	Advanced Solid Cancers	ADI PEG 20 + Pembrolizumab	Phase I	2017/7/14	33	25 patients were evaluated; 9 in the dose-escalation cohort and 16 in the expansion cohort. Mean arginine levels were suppressed for 1–3 weeks but gradually increased. CD3+ T cells increased in 10 of 12 subjects (83.3%) after ADI-PEG 20 treatment, including 3 PR (*p* = 0.02). PD-L1 expression was low and increased in 3 of 10 subjects (30%). 6 of 25 heavily pretreated patients had PR, including both ASS1 proficient and deficient subjects, for a response rate of 24%.	NCT03254732	[117]
Ph 2/3 Study in Subjects with MPM to Assess ADI-PEG 20 With Pemetrexed and Cisplatin	Completed	MPM	ADI-PEG 20 + Pemetrexed + Cisplatin	Phase II/III	2017/8/1	249	The plasma arginine decreased while citrulline increased and was maintained over 18 weeks of ADIPemCis therapy. In 31 pilot evaluable patients, the DCR was 93.5% (n = 29/31; 95% CI: 78.6–99.2%), with a PR rate of 35.5% (n = 11/31; 95% CI: 19.2–54.6%). The median PFS and OS were 5.6 (4.0–6.0) and 10.1 (6.1–11.1) months, respectively. The treatment was well tolerated.	NCT02709512	[112,118]
ADI-PEG 20 in Combination with Gemcitabine and Docetaxel for the Treatment of Soft Tissue Sarcoma, Osteosarcoma, Ewing’s Sarcoma, and Small Cell Lung Cancer	Completed	Sarcoma	ADI-PEG 20 + Gemcitabine + Docetaxel	Phase II	2018/5/9	98	75 patients were evaluated with PFS/OS (months) results of 6.0/N.D. for the 600 mg/m^2^ group (n = 31), 7.2/22.5 for leiomyosarcoma (LMS) (n = 33), 5.1/17.4 for liposarcoma, and 2.8/15.0 for other (n = 36). Out of 75 patients, 8% (6/75) had CR (3 LMS, 1 synovial, and 2 angiosarcoma), 17% (13/75) had PR, and 43% (32/75) had SD, leading to an ORR of 25% (19/75) and a clinical benefit rate of 68% (51/75). There was a tendency for ASS1 negative tumors to benefit more than ASS1 positive tumors.	NCT03449901	[119]
Study in Patients With Tumours Requiring Arginine to Assess ADI-PEG 20 With Atezolizumab, Pemetrexed and Carboplatin	Withdrawn	NSCLC	Atezolizumab + Pemetrexed + Carboplatin + ADI PEG 20	Phase I	2018/6/1	0	NA	NCT03498222	
Study of Immunotherapy Plus ADI-PEG 20 for the Treatment of Advanced Uveal Melanoma	Completed	Melanoma	ADI PEG 20 + Nivolumab + Ipilimumab	Phase I	2019/4/16	9	NA	NCT03922880	
ADI-PEG 20 Plus Radiotherapy and Temozolomide in Subjects with Glioblastoma Multiforme	Recruiting	GBM	ADI-PEG 20 + Temozolomide	Phase I	2020/9/14	Estimated 32	NA	NCT04587830	
Study of ADI-PEG 20 Versus Placebo in Subjects with Genotype WWOX-GG, Unresectable HCC	Recruiting	HCC	ADI-PEG 20	Phase III	2022/3/14	Estimated 150	NA	NCT05317819	
Study of ADI-PEG 20, Venetoclax and Azacitidine in Acute Myeloid Leukemia	Recruiting	AML	ADI-PEG 20	Phase I	2022/4/5	Estimated 60	NA	NCT05001828	
Nivolumab and ADI-PEG 20 Before Surgery for the Treatment of Resectable Liver Cancer	Withdrawn	Resectable HCC	Nivolumab + ADI-PEG 20 + Resection	Phase II	2022/4/13	0	NA	NCT04965714	
ADI-PEG 20 in Combination with Gemcitabine and Docetaxel After Progression on Frontline Therapy in Non-small Cell and Small Cell Lung Cancers	Not yet recruiting	NSCLC and SCLC	ADI-PEG 20 + Gemcitabine + Docetaxel	Phase I/II	2023/3/31	Estimated 108	NA	NCT05616624	
Study of ADI-PEG 20 or Placebo Plus Gem and Doc in Previously Treated Subjects with Leiomyosarcoma (ARGSARC)	Not yet recruiting	Sarcoma	ADI PEG 20	Phase III	2023/6/15	Estimated 300	NA	NCT05712694	

Abbreviation: HCC, hepatocellular carcinoma; SCLC, small cell lung cancer; NSCLC, non-small cell lung cancer; MPM, malignant pleural mesothelioma; OC, ovarian cancer; NHL, non-Hodgkin’s lymphoma; AML, acute myeloid leukemia; GBM, glioblastoma multiforme; GI cancer, gastrointestinal cancer; TACE, transarterial chemoembolization; mFOLFOX6, modified FOLFOX6; OS, overall survival; PFS, progression-free survival; TTP, time-to-tumor progression; SD, stable disease; DCR, disease control rate; ORR, overall response rate; PR, partial response; CR, complete response.

Taken together, the clinical trials investigating ADI-PEG 20 have yielded encouraging results in treating various types of cancers, with manageable dosing and minimal side effects. Notably, a national multicenter phase II/III clinical trial for mesothelioma (NCT02709512) has met its endpoints, with significantly improved overall survival and progression-free survival, now seeking FDA approval. These developments suggest that ADI-PEG 20 could be an important therapeutic option for patients with mesothelioma and other cancers, pending further research and clinical trials.

### 3.2. rhArg1

Another strategy of arginine deprivation therapy is to utilize the enzyme arginase to convert arginine into ornithine and urea, leading to arginine deprivation in tumor cells [120]. To this end, a recombinant human arginase 1 (rhArg1) (E.C.3.5.3.1) was developed as a potential therapeutic agent for anticancer treatment [121]. The PEGylation of rhArg1 (rhArg1-PEG or BCT-100) was shown to improve its pharmacokinetic profile, increase the half-life and stability of rhArg1 in the body, and reduce immunogenicity, making it a more effective and safe therapeutic option for cancer patients [121]. Preclinical studies demonstrated the efficacy of rhArg1-PEG in inhibiting the growth of various tumor types that are dependent on arginine for growth and survival, including lung cancer, HCC, breast cancer, bladder cancer, prostate cancer, and melanoma. In subsequent clinical trials, rhArg1-PEG has shown promising results in combination with other therapies for the treatment of various cancers.

#### 3.2.1. Preclinical Evidence Supporting rhArg1 as a Potential Strategy for Anticancer Therapy

RhArg1-PEG has shown potential in treating glioma, according to preclinical studies. In one study, the drug was demonstrated to have selective cytotoxicity to all nine GBM cell lines tested, while human fetal glial cells were not sensitive [122]. Another study investigated the combination of arginine deprivation therapy and irradiation as a treatment strategy for GBM. Using rhArg1-PEG or arginine-free diets in vitro, it was found that rhArg1-PEG inhibited growth and cell-cycle progression, and reduced growth recovery in four different GBM cell line models, with a significant radiosensitization effect that was more pronounced in GBM cells with p53 loss of function [123]. Moreover, a recent study found that blocking arginine metabolism through cationic amino acid transporter 1 (CAT-1)-dependent arginine uptake inhibition or therapeutic depletion of arginine using rhArg1-PEG, delayed tumor development and prolonged murine survival in neuroblastoma cells, where arginase 2 (ARG2) drives neuroblastoma cell proliferation by regulating arginine metabolism. This study also revealed the immune–metabolic regulatory loop between tumor cells and infiltrating myeloid cells regulating ARG2 expression in vitro, which could be clinically exploited [124].

Several preclinical studies have shown that rhArg1-PEG may be effective in treating various lung cancer subtypes. In NSCLC cells, rhArg1-PEG induced autophagy as a cytoprotective mechanism, but inhibiting autophagy enhanced rhArg1-PEG-induced cytotoxicity and apoptosis, indicating that combining rhArg1-PEG with autophagy inhibitors may be a hopeful therapeutic approach for NSCLC [125]. In SCLC, rhArg1-PEG inhibited the growth of tumors that could not synthesize arginine, resulting in apoptosis, G1-cell-cycle arrest, and anticancer effects in both cell lines and xenograft models with low expression of ASS1 and ornithine transcarbamylase (OTC) [126]. RhArg1-PEG reduced cell viability in some lung adenocarcinoma cell lines, but the combination treatment of α-difluoromethylornithine (DFMO) and rhArg1-PEG significantly suppressed xenograft growth and increased median survival compared with the control [127]. High-endogenous expression of ARG2 in lung SCC xenografts may impede the anti-tumor effect of rhArg1-PEG, but the silencing of ARG2 resensitized H520 xenografts to rhArg1-PEG treatment, partially mediated through arginine depletion via G1 arrest and apoptosis [128]. In SCLC, silencing the CNTN-1 gene in rhArg1-PEG-resistant cells resensitized them to treatment and attenuated the EMT phenotype, and AKT inhibitor LY294002 reversed EMT progression by inhibiting the AKT-signaling pathway in resistant cells [129]. RhArg1-PEG was effective in SCLC patient-derived xenografts, and it also promoted the recruitment of CD8+ tumor-infiltrating lymphocytes, supporting further clinical investigations of rhArg1-PEG in solid tumors and its clinical combination with immune checkpoint inhibitors [130].

RhArg1-PEG has also shown encouraging results as an effective anticancer therapy in HCC. One study found that it effectively inhibited ASS1-positive HCC cells that lacked OTC expression, while another study showed its ability to deplete arginine and cause cell-cycle arrests in HCC cell lines. Profiling tumor gene expression of ASS1 and OTC before treatment could predict tumor response to arginine depletion with arginine-depleting enzymes [131]. Citrulline was also found to restore cell growth in HCC cells treated with rhArg1-PEG, suggesting a potential role for OTC in the arginine auxotrophy and rhArg1-PEG sensitivity of HCC cells [132]. Additionally, rhArg1-PEG retained over 90% of its native catalytic activity and was effective in depleting arginine in rats and cultured human liver cancer cell lines [121]. These results suggest that rhArg1-PEG has the potential to serve as a novel agent for HCC therapy and merits further investigation.

RhArg1-PEG is also an option for the treatment of lymphoma and leukemia, as shown by preclinical studies where it inhibited growth, induced cell-cycle arrest, and triggered apoptosis in NHL cells, while also inducing autophagy [133,134]. However, T-lineage acute lymphoblastic leukemia (T-ALL) cells may be resistant due to the protective effect of human mesenchymal stromal cells (hMSCs), which can be overcome by pre-treating hMSCs with vincristine [135]. In vivo animal-based studies have demonstrated that RhArg1-PEG depletes arginine, leading to reduced AML engraftment and proliferation, and acts synergistically with cytarabine to eliminate AML cells [136]. RhArg1-PEG also displays cytotoxicity to ALL blasts from patients in vitro and can exert synergistic effects with dexamethasone [137]. RNA-sequencing data suggest that RhArg1-PEG may provide a potential therapeutic approach for targeting arginine metabolism in ALL cells [138]. Furthermore, canavanine enhances antineoplastic effects in murine leukemic cells, and RhArg1-PEG abrogates cell growth and increases canavanine cytotoxicity [139].

Preclinical studies also demonstrated the potential of rhArg1-PEG as a promising agent for treating other types of cancers, although evidence is limited. In melanoma, rhArg1-PEG induced cell-cycle arrest and apoptosis in vitro with fewer safety concerns compared to ADI, and the mechanism behind its antiproliferative activity may provide insights for designing future combination therapies for clinical trials [140]. RhArg1-PEG also induced autophagy in melanoma cells, and combining it with chloroquine may be a potential therapy for malignant melanoma [141]. In pancreatic cancer, arginine depletion using rhArg1-PEG decreased migration, adhesion, and invasion of PANC-1 cells, mediated by autophagy and decreased activation of members of the Rho GTPase family [142]. RhArg1-PEG also reduced intracellular arginine levels, induced apoptosis, and upregulated autophagy-related genes in bladder cancer cell lines, while suppressing the AKT/mTOR-signaling pathway through ROS-mediated mechanisms [143]. In prostate cancer cells, rhArg1-PEG induced cytotoxicity and autophagy, suggesting an alternative cell death mechanism [144]. Similarly, rhArg1-PEG inhibited cell growth and induced autophagy in triple-negative breast cancer cells, with the potential of formulating combination therapy with autophagic inhibitors [145]. In ovarian cancer cells, rhArg1-PEG induced non-apoptotic cell death mediated by autophagy [146], and decreased cell migration and adhesion through the autophagy-mediated inhibition of RhoA’s role in regulating motility and adhesion [147]. Finally, rhArg1-PEG reduced cell viability in vitro and suppressed tumor growth in vivo in both 211H and H226 xenograft models of malignant pleural mesothelioma, while combining rhArg1-PEG with pemetrexed or cisplatin did not provide additional benefits [148].

Human arginase 1 mutant-PEG 20 (HAI-PEG 20) is a mono-PEGylated rhArg1 that effectively inhibits the growth of at least eight types of cancer cells in vitro and in vivo [149]. To produce this drug, researchers screened metal ions to create a site-specific mono-PEGylated human arginase I mutant conjugated to PEG-maleimide to produce a homogeneous product. Co^2+^ and Ni^2+^ enriched rhArg1 mutants demonstrated higher catalytic efficiency than wild type or Mn^2+^ enriched mutants, and the PEGylation did not affect enzyme activity or protein structures. Both Co-HAI-PEG20L and Ni-HAI-PEG20L inhibited the growth of eight types of cancer cell lines in vitro, and in mice, and maintained L-arginine levels below the detection limit for over 120 h without significant toxicity. While these findings suggest the potential of Co-HAI-PEG20L and Ni-HAI-PEG20L for anticancer therapy, their efficacy needs further evaluation in clinical trials [149].

#### 3.2.2. Overview of Clinical Trials Using rhArg1-PEG for Cancer Therapy and Their Outcomes

As of now, the National Clinical Trial database contains eight registered clinical trials utilizing rhArg1-PEG that are either ongoing or terminated (see Table 3). These studies primarily focus on evaluating the effectiveness of rhArg1-PEG for HCC, AML, ALL, melanoma, and prostate cancer, with limited investigation into other cancer types. The following sections describe and discuss the published findings from these trials.

RhArg1-PEG shows promise in treating HCC, with clinical trials demonstrating its safety and efficacy. In a phase I study, rhArg1-PEG induced dose-dependent arginine depletion, with an optimal biological dose of 1600 U/kg, leading to stable disease in 26.7% of patients [150]. In a subsequent phase II study, patients who had adequate arginine depletion for over two months had significantly longer progression-free survival times, and the drug was well-tolerated [151]. Another phase II study showed a disease control rate of 21.7% in chemo-naïve sorafenib-failure HCC patients, and suggested ASS1-negativity as a potential biomarker for overall survival [152]. RhArg1-PEG was also administered in combination with chemotherapy in pre-treated HCC patients, with good anti-cancer activity and a disease control rate of 36% [153]. Further studies are needed to evaluate its use in different patient populations and in combination with other agents.

Clinical trials were conducted to investigate the use of rhArg1-PEG for treating cancer types other than HCC. In a phase I study involving 23 patients with malignant melanoma and castration-resistant prostate cancer, the drug was well-tolerated, and sustained arginine depletion was observed with anti-tumor activity in both types of cancer. Two complete remissions and one partial response were observed, and a dose of 2.7 mg/kg/week was selected for future phase II studies [154]. Additionally, a 65-year-old patient with metastatic melanoma achieved complete remission lasting over 30 months after failing two immunotherapy strategies [155]. In AML, a randomized study of low-dose cytarabine with rhArg1-PEG versus low-dose cytarabine alone was conducted for AML patients who were not suitable for intensive therapy. Although the addition of rhArg1-PEG to low-dose cytarabine was well-tolerated, it did not result in a significant difference in the overall response rate or survival between the treatment arms [156].

While the use of rhArg1-PEG in treating cancer types other than HCC is still in the early stages, the results of clinical trials so far have been encouraging. The studies have shown that rhArg1-PEG is well-tolerated and has anti-tumor activities in both prostate cancer and melanoma, with some patients achieving complete remission. However, further studies are needed to determine the optimal dose and treatment duration of rhArg1-PEG, as well as to investigate its efficacy in combination with other therapies. Additionally, research is ongoing to identify patient subgroups who are most likely to benefit from rhArg1-PEG treatment based on molecular or cytogenetic findings. Overall, the results of these studies suggest that rhArg1-PEG has the potential to become an effective therapy for cancer patients who are not responding to other treatments.

**Table 3:** Summary of clinical trials evaluating therapeutic efficacy of rhArg1-PEG for cancer therapy.

Title	Status	Conditions	Interventions	Phases	Start Date	Enrollment	Outcome	NCT	Ref
Study of PEGylated Human Recombinant Arginase for Liver Cancer	Completed	HCC	rhArg1-PEG + Doxorubicin	Phase I	2008/5/1	15	NA	NCT00988195	
Study of PEGylated Human Recombinant Arginase for Liver Cancer (BCT-100-002)	Completed	HCC	rhArg1-PEG	Phase I/II	2010/3/1	20	May be included in NCT02089763.	NCT01092091	[152]
A Pilot Study of Recombinant Human Arginase 1 (rhArg1) in Patients with Relapsed or Refractory Leukemia or Lymphoma	Terminated	Leukemia and Lymphoma	rhArg1-PEG	Phase I	2012/4/1	1	NA	NCT01551628	
Efficacy Study of PEGylated Recombinant Human Arginase 1 as a Second-line Therapy in Patients with Advanced Liver Cancer	Completed	HCC	rhArg1-PEG	Phase II	2014/4/1	27	27 patients were recruited, with a median TTP and PFS of 6 (95% CI, 5.9–6.0) weeks and a DCR of 21.7% (5 SD). The drug was well tolerated. Duration of arginine depletion correlated with OS. Among patients with available IHC results, those with ASS1-negative tumors had an OS of 35 (95% CI: 8.3–78.0) weeks, compared to 15.14 (95% CI: 13.4–15.1) weeks in those with ASS1-positive tumors.	NCT02089763	[152]
PEGylated Recombinant Human Arginase 1 in Combination with Oxaliplatin and Capecitabine for the Treatment of HCC	Completed	HCC	rhArg1-PEG + Oxaliplatin + Capecitabine	Phase II	2014/4/1	17	17 patients received oxaliplatin at 3 dose levels: 85 mg/m^2^ (8 patients), 100 mg/m^2^ (3 patients), and 130 mg/m^2^ (6 patients), with no dose-limiting toxicity. Median study duration was 8 weeks. Among 14 evaluable cases, one achieved PR, 4 had SD, and the DCR was 36%. Most responses occurred in the 130 mg/m^2^ cohort with 1 PR and 2 SD. Median TTP and PFS were both 7.0 weeks. Overall median OS was 10.7 months, and median OS was not reached at 19.4 months of follow-up in the 130 mg/m^2^ cohort.	NCT02089633	[153]
Recombinant Human Arginase 1 (rhArg1) in Patients with Advanced Arginine Auxotrophic Solid Tumors	Completed	Melanoma and Prostate cancer	rhArg1-PEG	Phase I	2014/11/1	23	A 65-year-old patient with metastatic melanoma, who failed two immunotherapy strategies, received 2 mg/kg intravenously weekly BCT-100. The patient had no toxicities > grade 2 and achieved CR for over 30 months. The tumor lacked expression of ASS1 and OTC.	NCT02285101	[155]
Efficacy and Safety Study of Recombinant Human Arginase 1 in Patients with Relapsed or Refractory Acute Myeloid Leukemia	Unknown	AML	rhArg1-PEG	Phase II	2016/9/1	Estimated 25	NA	NCT02899286	
A Study Evaluating the Safety and Activity of PEGylated Recombinant Human Arginase (BCT-100)	Completed	AML and ALL	rhArg1-PEG	Phase I/II	2018/8/28	49	NA	NCT03455140	

Abbreviation: HCC, hepatocellular carcinoma; AML, acute myeloid leukemia; ALL, acute lymphoblastic leukemia; OS, overall survival; PFS, progression-free survival; TTP, time-to-tumor progression; SD, stable disease; DCR, disease control rate; PR, partial response; CR, complete response.

### 3.3. rhADC

A different method of arginine deprivation therapy involves the use of rhADC (E.C.4.1.1.19), an enzyme that breaks down arginine into carbon dioxide and agmatine, which is then metabolized into putrescine and spermidine—two polyamines essential for cell survival and proliferation [157]. Agmatine inhibits the proliferation of tumor cells and reduces intracellular polyamine content [158]. Agmatine-induced inhibition of cell proliferation promotes apoptosis, and the depletion of arginine by applying rhADC to tumor cells may inhibit their growth [157,158]. Evidence has demonstrated the effectiveness of rhADC in inhibiting cancer cell growth and inducing cell death since 2003 [159]. However, the development of rhADC may have faced challenges related to efficacy, safety, and cost, which could have slowed down progress in this area of research. It is also possible that research in this area is still ongoing but has not been widely publicized [160]. Further research is needed to fully understand the current status of rhADC as a potential anticancer therapy.

### 3.4. BCA

A new arginase enzyme, BCA, which is produced by the bacterium Bacillus Caldovelox, isolated from a deep-sea hydrothermal vent, was recently characterized. Studies have investigated the anticancer effects of combining dichloroacetate with BCA or rhArg1 on breast cancer cells and xenograft models, showing significant anti-proliferative effects on MCF-7 and MDA-MB 231 cells and enhanced anti-tumor activity in an MDA-MB 231 xenograft mouse model [161]. In addition, BCA-M, a mutant of BCA, was shown to inhibit the growth of cervical cancer cells in vitro and induce apoptosis and cell-cycle arrest, with autophagy serving as a protective mechanism [162]. Furthermore, the therapeutic efficacy of BCA-M was improved by developing a thermostable arginine-depleting enzyme, BCA-M-PEG20, which significantly inhibited the growth of ASS-positive lung cancer cells and was well-tolerated in mouse models [163]. BCA-M-PEG20 demonstrated efficacy in gastric cancer treatment by inhibiting the growth of ASS-positive gastric cancer cells and suppressing tumor growth in a MKN-45 gastric cancer xenograft model [164]. These findings suggest the potential of BCA-M and BCA-M-PEG20 as anticancer therapies, but further investigations involving clinical trials are necessary for their full potential to be understood.

## 4. Potential Strategies for Arginine Deprivation Therapy in Cancer Independent of Arginine-Depleting Enzymes

Apart from enzyme-mediated arginine depletion, there are several other approaches to deprive arginine that could be formulated as new anticancer treatments. One possible approach is to target the transport mechanisms responsible for importing arginine into the cell. Small molecule inhibitors can target system L-amino acid transporter (LAT1) and CAT-1, both of which are involved in the transport of arginine into the cell. Inhibiting these transporters was shown to induce cytotoxicity in cancer cells that rely on arginine for survival [165,166,167].

Another approach is to target upstream regulators of the arginine metabolic pathway, such as the activating transcription factor-4 (ATF4). ATF4 plays a crucial role in the induction of arginine metabolism in response to amino acid deprivation and can be activated upon arginine deprivation. The inhibition of ATF4 was shown to induce cell death in arginine-dependent cancer cells [17]. Although most of the proposed strategies to target ATF expression or function are indirect, such as the use of BRAF kinase inhibitors [168], these approaches show potential and warrant further investigation.

Moreover, targeting the mTORC1 pathway was also shown to induce arginine deprivation by inhibiting the expression of ASS1 and other enzymes involved in the arginine metabolic pathway. Arginine is one of the amino acids that can directly regulate mTORC1 through the cytosolic arginine sensor for mTORC1 subunit 1 (CASTOR1) [169]. However, prolonged deprivation of arginine or leucine induces PI3K/Akt-dependent reactivation of mTORC1 [170]. It is noteworthy that some cell types with high CASTOR1 expression were shown to be insensitive to arginine regulation [171]. Therefore, targeting the mTORC1 pathway may also contribute to an arginine-deprivation-like phenotype in cancer cells, suggesting the potential for combining arginine-depleting enzymes and mTORC1 targeting agents in future clinical trials.

One strategy to enhance the anticancer effects of arginine deprivation is to use an arginine analog, canavanine, which is a plant-derived natural compound [172]. Arginine deprivation induces prolonged endoplasmic reticulum (ER) stress and activation of the unfolded protein response, which activates Akt- and MAPK-dependent pathways to counteract proapoptotic signaling. However, treatment with canavanine can critically augment ER stress and apoptosis induction in arginine-starved cells, potentially enhancing the efficacy of arginine deprivation-based anticancer therapy [173]. In head and neck squamous cell carcinoma cells, arginine deficiency induces global metabolic changes, protein/membrane breakdown, and specific ER stress responses that differ among cells with different intrinsic arginine deprivation sensitivity. The combination of arginine deprivation with canavanine induces catastrophic ER stress via the eIF2α-ATF4(GADD34)-CHOP pathway, leading to apoptosis. This pathway is not involved in arginine deprivation-related radiosensitization, suggesting that ER stress pathways could be a novel target for improving multi-modal metabolic anticancer therapy [174]. Moreover, canavanine enhances antineoplastic effects in murine leukemic cells, and RhArg1-PEG abrogates cell growth and increases canavanine cytotoxicity [139]. Thus, the combination of arginine deprivation with canavanine and other targeted therapies that induce ER stress may hold great promise for improving anticancer therapy.

## 5. Biomarkers for Arginine Deprivation Therapy in Cancers

**Figure 3:**
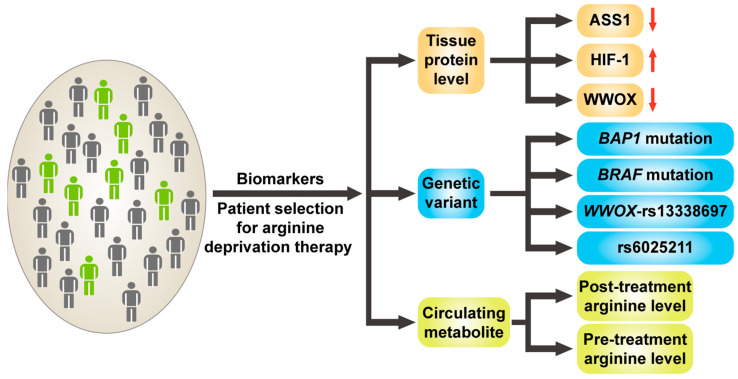
The diagram highlights potential biomarkers that can aid in patient selection for arginine deprivation anticancer therapy. These biomarkers encompass lower levels of ASS1 and WWOX proteins in tissues (red arrow pointing down), in addition to increased expression of HIF-1 protein (red arrow pointing up). Furthermore, genetic variations, including mutations in the *BAP1* and *BRAF* genes, as well as single nucleotide polymorphism (SNP) variations, specifically *WWOX*-rs13338697 and rs6025211, may also serve as biomarkers. Additionally, pre-treatment and post-treatment circulating arginine levels can be used to predict the therapeutic efficacy of this therapy for cancer treatment. Patients highlighted in gray may not respond to arginine deprivation therapy, while those in green may potentially have a positive response to this therapy. Abbreviation: ASS1, arginosuccinate synthetase 1; HIF-1, hypoxia-inducible factor-1, WWOX, WW-domain-containing oxidoreductase; BAP1, BRCA1 associated protein 1; BRAF, B-Raf proto-oncogene, serine/threonine kinase.

Despite the encouraging results of arginine deprivation therapy, there remains a high degree of variability in patients’ responses, with the reasons largely unknown. It is therefore essential to identify biomarkers that can predict the efficacy of this therapy in individual patients prior to treatment, thereby preventing unnecessary side effects and/or the delay of optimal treatments in non-responders. Currently, several potential biomarkers have been proposed, including levels of tumor proteins such as ASS1, HIF-1, and WWOX. Certain genetic variations, such as specific single nucleotide polymorphisms (SNPs), were proposed, as well as the levels of some circulating metabolites [175] (Figure 3). In the following sections, these potential biomarkers are described and discussed.

### 5.1. Tissue protein Level

#### 5.1.1. ASS1

Studies have also shown that low ASS1 expression is associated with poor prognosis and resistance to conventional chemotherapy in various types of cancer, indicating that alternative therapeutic strategies such as arginine deprivation therapy may be necessary for these patients [21,34,52,54,176].

A preclinical study investigated the role of ASS1 as a biomarker to predict response to ADI-PEG 20 in lymphoid malignancies. It was found that ASS1 promoter methylation was frequent in malignant lymphoid tissues and low ASS1 expression was associated with sensitivity to ADI-PEG 20 [36]. The study also demonstrated that the demethylating agent 5-Aza-dC reactivated ASS1 expression and rescued lymphoma cell lines from ADI-PEG 20 cytotoxicity. Furthermore, ASS1-methylated cell lines exhibited autophagy and caspase-dependent apoptosis following treatment with ADI-PEG 20, and the autophagy inhibitor chloroquine potentiated the apoptotic effect of ADI-PEG 20 in malignant lymphoid cells and patient-derived tumor cells.

A phase I clinical trial investigated the predictive value of ASS1 expression in melanoma tumors for response to arginine-depleting therapy using ADI-PEG 20 [106]. The results showed that patients with negative ASS1 expression had a higher clinical benefit rate and a longer time to progression compared to patients with positive ASS1 expression, suggesting that ADI-PEG 20 is only effective in melanoma patients whose tumors have negative ASS1 expression. Conversely, ASS1 tumor positivity was associated with drug resistance and tumor progression.

Therefore, ASS1 expression levels in tumor tissues may serve as a predictive biomarker in the selection of patients who are more likely to benefit from arginine deprivation therapy. Low ASS1 expression is associated with poor prognosis and resistance to conventional chemotherapy in various types of cancer. The sensitivity to arginine deprivation therapy is inversely correlated with ASS1 expression levels in preclinical studies and clinical trials.

#### 5.1.2. HIF-1

HIF-1 is a transcription factor that plays a crucial role in the cellular response to hypoxia. Apart from its involvement in oxygen homeostasis, HIF-1 also participates in numerous cellular processes such as metabolism, angiogenesis, and apoptosis, all of which are critical in cancer development and progression. It has been found that HIF-1 expression is upregulated in different types of cancers and that this upregulation is associated with increased tumor growth, invasion, and metastasis. HIF-1 has also been shown to play a role in regulating arginine metabolism in cancer cells [177]. As a result, HIF-1 renders cancer cells more resistant to chemotherapy and radiation therapy and, at the same time, more reliant on exogenous sources of arginine [178].

In preclinical studies using human cancer cell lines, it was demonstrated that arginine deprivation therapy reduces HIF-1α and HIF-2α protein levels [179]. This indicates that HIF-1 expression levels might serve as an on-therapy biomarker for predicting responses to arginine deprivation therapy in cancer. Additionally, it was proposed that tumors with high HIF-1 expression levels may be more sensitive to arginine deprivation therapy than those with low HIF-1 expression levels [28,179]. This is because HIF-1 upregulation is associated with increased arginine consumption (due to ASS1 inhibition) in cancer cells. Therefore, the inhibition of arginine metabolism by arginine deprivation therapy may have a greater effect on HIF-1-expressing tumors. In a preclinical study using HCC cell lines, it was observed that sensitivity to arginine deprivation therapy with ADI-PEG 20 is positively correlated with levels of HIF-1α expression. Cells with high HIF-1α expression were more sensitive to arginine deprivation therapy than those with low HIF-1α expression [28].

The findings imply that the expression levels of HIF-1 have the potential to serve as predictive biomarkers for arginine deprivation therapy in cancer. The findings also suggest that tumors with elevated HIF-1 expression levels may display a heightened sensitivity to this treatment. Nonetheless, due to the intricate relationship between HIF-1 expression and responses to arginine deprivation therapy, which may be influenced by factors such as tumor type, genetic background, and environmental factors, additional research, including clinical trials, are necessary to validate these findings.

#### 5.1.3. WWOX

WWOX is a crucial tumor-suppressor gene involved in various cellular processes such as DNA repair, cell-cycle regulation, apoptosis, and transcriptional regulation [180]. Recent studies demonstrated that WWOX plays a pivotal role in the cellular response to hypoxia, a hallmark of the tumor microenvironment [180]. Several studies indicated that WWOX expression is reduced in hypoxic conditions in cancers. This downregulation of WWOX expression is linked to increased cell survival, proliferation, migration, and invasion, promoting tumor progression and metastasis [181,182,183].

HIF-1α was identified as a critical mediator of hypoxia-induced WWOX downregulation in HCC cells. HIF-1α binds to the WWOX-promoter region and represses WWOX expression in a hypoxia-dependent manner. Knockdown of HIF-1α restores WWOX expression and inhibits cell proliferation and migration in HCC cells under hypoxic conditions [183]. In breast cancer cells, WWOX also plays a vital role in regulating hypoxia-mediated autophagy. Reduced WWOX expression under hypoxic conditions activates the AMPK pathway, leading to autophagy induction, where autophagy exerts pro-metastatic or anti-metastatic functions dependent on cancer stages. Targeting the WWOX–AMPK axis may be a potential therapeutic strategy for breast cancer treatment [184]. These studies highlight the key regulatory role of WWOX in the cellular response to hypoxia, and the hypoxia-induced downregulation of WWOX expression is associated with tumor progression and metastasis.

Recent research has evaluated WWOX expression as a potential biomarker in the response to ADI-PEG 20 treatment in HCC cells. The study found that low levels of WWOX expression, indicative of hypoxic conditions, were associated with a better response to ADI-PEG 20 treatment [28]. These findings suggest that WWOX expression may be a useful biomarker in predicting the response to arginine deprivation therapy in HCC patients, particularly when combined with the status of hypoxia and the tissue levels of HIF-1.

### 5.2. Genetic Biomarker

Numerous studies have reported on the potential of biomarkers for arginine deprivation therapy. For instance, one study investigated the effects of nuclear deubiquitylase BRCA1-associated protein 1 (BAP1) deficiency on MPM cell physiology and drug sensitivity [81]. As BAP1 is commonly inactivated in MPM and its germline mutation is linked to MPM and other cancers, this study found that BAP1-deficient cells experienced changes in arginine metabolism, leading to increased ASS1 expression and altered sensitivity to arginine deprivation by ADI-PEG 20. Consequently, this study suggests that a specific germline mutation within the BAP1 gene could identify epithelioid MPM patients who may benefit from ADI-PEG 20 treatment [81].

Moreover, melanomas with the V600E mutation are frequently treated with BRAF inhibitors (BRAFi). However, BRAFi-resistant melanomas are susceptible to arginine deprivation due to their inability to re-express ASS1 and by causing ineffective autophagy, where autophagy promotes melanoma tumor progression [185,186]. Mechanistically, the downregulation of deubiquitinase USP28 or AMPK-α1 in BRAFi-resistant cells was identified as the primary mechanism responsible for the inability to re-express ASS1 upon arginine deprivation. Therefore, combining arginine deprivation with USP28 overexpression or the overexpression of autophagy-associated proteins, such as Atg5 or AMPK-α1, may redirect arginine deprivation-induced apoptosis toward autophagy and enhance the therapeutic efficacy of this approach, providing a potential therapeutic option for BRAFi-resistant melanoma patients.

In a recent study, the association between three SNPs and therapeutic outcomes in advanced HCC patients treated with ADI-PEG 20 was examined [28]. The study found that the *WWOX*-rs13338697-GG genotype was associated with favorable overall survival, whereas the rs6025211-TT genotype was associated with unfavorable time-to-tumor progression. Thus, the genotypes of these two SNPs are considered as potential genetic biomarkers. Mechanistically, it was found that the *WWOX*-rs13338697-GG genotype was associated with lower tissue WWOX and ASS1 levels and higher pretreatment plasma arginine levels. The silencing of WWOX in HCC cells enhanced HIF1A increments under hypoxia, further decreased ASS1, and increased cell susceptibility to ADI-PEG 20.

While these findings suggest potential genetic biomarkers for the prediction of therapeutic responses in patients with specific cancer types, further investigation is needed. Currently, a phase III clinical trial recruiting HCC patients with the *WWOX*-rs13338697-GG genotype and using a ADI-PEG 20 monotherapy is underway (NCT05317819).

### 5.3. Plasma Biomarker

The use of blood metabolites as biomarkers to predict the efficacy of arginine deprivation therapy in cancer is a promising avenue of research [116,150]. The current literature suggests that the levels of arginine-related metabolites, such as arginine, citrulline, and/or ornithine, could serve as potential therapeutic efficacy biomarkers during the on-treatment phase [116,150]. It was observed that a constitutive reduction in arginine with increases in citrulline and/or ornithine levels is associated with better treatment outcomes [116,150].

Interestingly, while previous studies have primarily focused on on-treatment blood circulating levels of arginine, citrulline, and/or ornithine, recent research has shown that pre-treatment levels of these metabolites may also be associated with the therapeutic efficacy of arginine deprivation therapy [28]. For instance, HCC patients with the *WWOX*-rs13338697-GG genotype, which is linked to better therapeutic outcomes, also had higher pre-treatment circulating arginine levels [28]. Studies indicated that the HCC harboring the *WWOX*-rs13338697-GG genotype had lower WWOX and ASS1 expressions, constituting an arginine-auxotrophic phenotype. Such cells require high serum arginine supply to survive and are therefore more sensitive to arginine deprivation therapy. As such, one can assume that patients with high serum arginine are prone to carrying cancers with arginine-auxotrophic phenotypes. Selecting patients with high pre-treatment circulating arginine levels may serve as a potential biomarker for arginine deprivation therapy in the future.

However, further clinical studies are needed to validate these potential biomarkers and determine their clinical utility in predicting the efficacy of arginine deprivation therapy in cancer. This will require large-scale studies that investigate the association between blood metabolite levels and treatment outcomes in different types of cancer and patient populations.

## 6. Conclusions and Future Perspective

Based on the current understanding and findings, arginine deprivation therapy offers several advantages over conventional chemotherapy. Table 4 provides a summary of the benefits and challenges associated with this therapy, highlighting its lower toxicity, reduced side effects, good tolerability, and potential as an add-on therapy. However, it is important to note the effectiveness of this therapy is limited to individuals with specific biomarkers, such as being ASS1-negative. These enzyme-drugs are still in the early stages of development and can potentially be modified to achieve different administration routes and different cellular targets. Given its low toxicity, this therapy can also be used in combination with other cancer treatments, such as radiation therapy, immunotherapy, or chemotherapy, to enhance its overall effectiveness as an add-on therapy.

Presently, arginine deprivation therapy can be achieved with arginine-degrading enzymes, including arginine deiminase, arginase, and arginine decarboxylase. Preclinical studies demonstrated the significant antitumor activities of arginine deprivation therapy in various type of cancers, such as melanoma, hepatocellular carcinoma, mesothelioma, and others. In a randomized clinical trial, arginine deprivation therapy showed efficacious results in patients with advanced mesothelioma, with improved progression-free survival and overall survival compared to the standard of care. At present, clinical trials for arginine deprivation therapy on many different cancers are vigorously undergoing.

Future research in arginine deprivation therapy should be focused on identifying biomarkers that can predict patients’ responses to treatments. It is also important to optimize the treatment schedules and dosages in combination treatments, so as to reduce the side effects of chemotherapy, radiotherapy, and immunotherapy. Efforts are also being made to develop more potent and specific arginine-degrading enzymes and analogs for arginine deprivation.

Overall, arginine deprivation therapy holds great promise as a targeted therapy for cancer treatment, and further research is needed to fully understand its potential and to optimize its use in cancer clinics.

## Figures and Tables

**Table 1:** Overview of cancer types exhibiting low ASS1 expression and suitable for arginine deprivation therapy.

Cancer Type	ASS1 Expression	Method	Reference
Glioma	Reduced in tumor	IHC and WB	[23,24]
Lung cancer	Reduced in SCLC and LCNEC	IHC	[25,26]
Head and neck cancer	Reduced in tumor	IHC	[27]
Hepatocellular carcinoma	Reduced in tumor	cDNA dot blotting, IHC and WB	[21,28]
Pancreatic cancer	Reduced in tumor	IHC and cDNA dot blotting	[21,29]
Renal cancer	Reduced in tumor	IHC and cDNA dot blotting	[21,30]
Bladder cancer	Reduced in tumor	IHC and RT-qPCR	[31,32]
Prostate cancer	Reduced in tumor	cDNA dot blotting	[21]
Breast cancer	Reduced in tumor	RT-qPCR	[33]
Sarcoma	Reduced in tumor	cDNA microarray and IHC	[34]
Ovarian cancer	Reduced in tumor	cDNA dot blotting and IHC	[21,35]
Melanoma	Reduced in tumor	cDNA dot blotting	[21]
Lymphoma and leukemia	Reduced in tumor	IHC, WB and RT-qPCR	[36,37]
Mesothelioma	Reduced in tumor	cDNA dot blotting	[21]

IHC, immunohistochemical staining; WB, western blotting; RT-qPCR, real-time quantitative PCR.

**Table 4:** The benefits and disadvantages of the arginine deprivation therapy.

Benefit	Disadvantage and Challenge
Lower toxicity	Only effective in individuals with specific biomarkers (e.g., ASS1-negative)
Fewer side effects	
Good tolerability	
Potential add-on therapy	

## Data Availability

Not applicable.
